# Acupuncture plus massage for cervicogenic headache: A protocol for systematic review and meta-analysis

**DOI:** 10.1097/MD.0000000000028736

**Published:** 2022-01-28

**Authors:** Fangfang Ding, Zhen Liu, Rui Li, Chenying Wang, Yan Lu

**Affiliations:** Department of Acupuncture and Massage, Shandong University of Traditional Chinese Medicine, Shandong, China.

**Keywords:** acupuncture, cervicogenic headache, massage, protocol, systematic review

## Abstract

**Background:**

Cervicogenic headache (CGH), one of the most common headaches. It is characterized by pain starting from the neck and gradually involving the eyes, frontal and temporal regions. Acupuncture and massage therapy has been widely used in the treatment of CGH, the purpose of this study was to evaluate the effectiveness and safety of acupuncture combined with massage in the treatment of CGH.

**Methods:**

The databases of PubMed, Embase, Cochrane library, Medicine, Web of Science, China National Knowledge Infrastructure, Wan-Fang Database, China Biomedical Literature Service System, and Chongqing VIP Chinese Science will be searched. This study will include randomized controlled trials of acupuncture combined with massage in the treatment of CGH published before November 2021. The primary outcomes included the overall efficiency, visual simulation score, neck activity score, quality of life score, and adverse reactions as secondary outcomes were assessed. Cochrane bias risk assessment tool will be used for literature data screening and quality evaluation, and using RevMan5.4 to collect data for statistical analysis. We then will use the Grading of Recommendations Assessment, Development, and Evaluation approach to assess the overall quality of evidence supporting the primary outcomes.

**Results:**

This systematic review will provide a high-quality synthesis to evaluate the efficacy and safety of acupuncture combined with massage therapy in the treatment of CGH, providing a reference for the safe and effective treatment plan.

**Conclusion:**

This study provides evidence that acupuncture combined with massage is effective and safe for patients with CGH.

**Systematic review registration:**

INPLASY2021120049U1.

## 1 Introduction

Cervicogenic headache (CGH) is defined as any headache caused by disturbances of the cervical spine or its components (such as bone, disc, and/or soft tissue composition), usually but not always accompanied by neck pain.^[1]^ Its incidence rate accounts for 1.0% to 4.1%. Data have revealed that it even ranged to 17.5% in patients with severe headache.^[2,3]^ The clinical manifestations indicate that pain starting in the neck, which can gradually involve the ocular, frontal, and temporal areas, partly accompanied by nausea, vomiting, numbness, and discomfort in the head, neck, or upper limbs,^[4,5]^ study^[6]^ shows that neck dysfunction may be the main cause of CGH patients, in which neck joint problems, neck muscle and fascia problems can be induced.

At present, conservative therapies for CGH mainly include nerve block therapy, non-steroidal anti-inflammatory drugs, corticoid pulse radiofrequency therapy, acupuncture therapy, massage therapy, rehabilitation therapy, and other therapies.^[7,8]^ Among them, acupuncture and massage as a part of traditional Chinese medicine therapy have gradually become the research hotspot^[8]^ of treating this disease because of their advantages of loosening adhesion, effectively relieving muscle spasm, reducing pain and local pressure, and small side effects. Multiple traditional studies^[9–12]^ have confirmed the significant clinical efficacy of acupuncture or massage in CGH, based on this, we explore whether acupuncture combined with massage has better efficacy compared with other therapies. In this study, the purpose of this systematic review was to assess the effectiveness and safety of acupuncture combined with massage for CGH and to provide evidence for clinical decision-making.

## 2 Methods and analysis

This protocol was conducted following the guidelines of the Preferred Reporting Items for Systematic Review and Meta-Analysis Protocol (PRISMA-P).^[13]^ It was registered on the international platform of registered systematic review and meta-analysis protocols (INPLASY2021120049U1).

### 2.1 Inclusion criteria

#### 2.1.1 Types of studies

1. **Inclusion:** This study will include only randomized controlled trials combining acupuncture and massage for CGH.
2. **Exclusion:** The types of repeated published literatures or non-randomized controlled trials, such as disease cases, animal trials, reviews, expert experience elaborations, systematic evaluations will be excluded.

#### 2.1.2 Types of participants

All adult patients with clinical diagnosis and clear efficacy criteria of CGH will be included in this study. However, location age, gender, and duration of patients are not limited. The diagnosis should refer to the criteria of the Efficacy Standards for the Diagnosis of Traditional Chinese Medicine or the diagnostic criteria of CGH formulated by the International Headache Association.

#### 2.1.3 Types of interventions

The treatment intervention must be acupuncture combined with massage, but the acupoints, frequency, and techniques of acupuncture or massage were not limited. The intervention of control group is other effective treatments for CGH which is different from acupuncture combined massage, such as simple western medicine treatment or simple acupuncture treatment.

#### 2.1.4 Types of outcomes

We will consider the following indexes: the overall efficiency, the visual simulation score, neck activity score, and quality of life score as the primary outcomes. In addition, we will record the adverse reactions and abscission of patients during the treatment.

### 2.2 Data sources and search methods

#### 2.2.1 Electronic searches

We will collect relevant articles by searching the following databases: PubMed, Web of Science, Medicine, EMBASE, Cochrane Library, Wan-Fang Database, China National Knowledge Infrastructure, China Science Journal Database, and China Biomedical Literature Database. We will select the studies which is published up to November 2021. The retrieval method is combined mesh words with free words, and the retrieval words included: acupuncture therapy, acupuncture, acupuncture points, warm acupuncture, fire needling, acupuncture analgesia, tuina, massage, manipulation, CGH, cervical headache, randomized controlled trial, case control studies and trials, etc. The search strategy for the PUBMED is presented in Table 1.

**Table 1:** Search strategy for the PubMed database.

Number	Terms
#1	Acupuncture Therapy [MeSH]
#2	Acupuncture [MeSH]
#3	Ear Acupunctures [MeSH]
#4	Acupuncture Points [MeSH]
#5	Acupuncture Analgesia [MeSH]
#6	Dry Needling [MeSH]
#7	Electroacupuncture [MeSH]
#8	Fire needling [all field]
#9	Scalp acupuncture [all field]
#10	Intradermal needling [all field]
#11	Catgut embedding [all field]
#12	#1 or #2–11
#13	Massage[MESH]
#14	Chiropractic Adjustment [MeSH]
#15	Tuina [all field]
#16	Knead [all field]
#17	Manipulation [all field]
#18	#13 or #14–17
#19	Cervicogenic Headache [MeSH]
#20	Post-Traumatic Headache [MeSH]
#21	#20 or #21
#22	Randomized controlled trial [MeSH]
#23	Trial [MeSH]
#24	Case control studies [MeSH]
#25	Observational studies [MeSH]
#26	#22 or #23–25
#27	#12 and #18 and #21 and #26

#### 2.2.2 Searching for other resources

We will search a reference list for identifying published journals, books, conference articles, and gray literature related to this research topic. We will retrieve manually related documents, such as replacing and supplementing some reference documents, such as medical textbooks and clinical laboratory manuals to make our search as complete as possible.

### 2.3 Data collection and export

**Figure 1:**
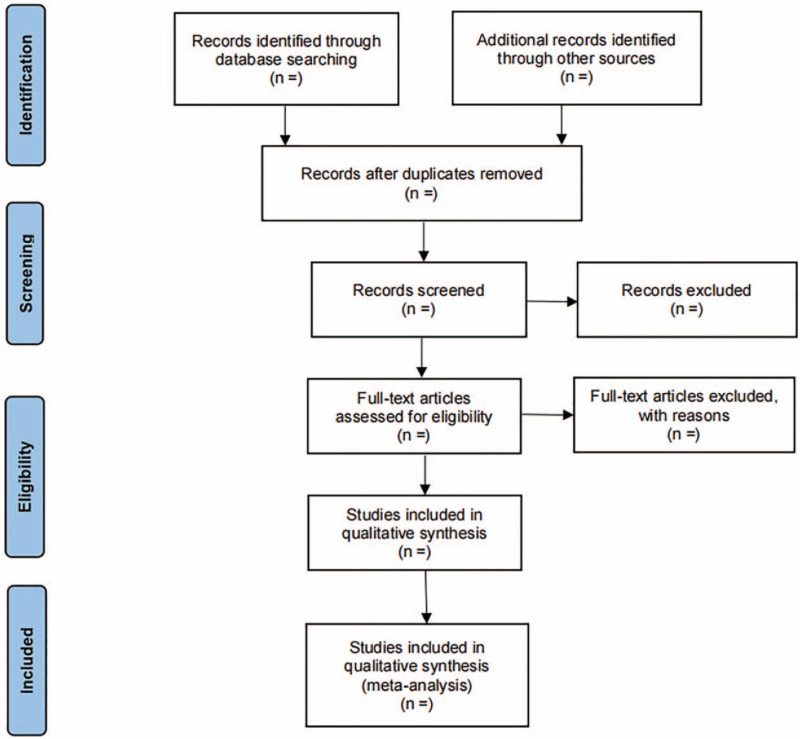
Flow chart of literature selection process.

Relevant literature was independently searched and screened by 2 researchers, first removing duplicates studies by the software EndNote (version X9, Clarivate Analytics), excluding apparently irrelevant literature by reading questions and abstracts, then reading the full text, excluding ineligible literatures again according to the inclusion criteria, and recording the reasons for the deletion. Cross-check the information, and finally be determined by the third researcher in case of differences. A flowchart of the screening process is presented in Figure 1.

### 2.4 Data extraction and analysis

We will use Excel recording the basic information to find an extraction table, the basic information included: title, year of publication, the first author, country, study cases of treatment group and the control group, disease course, intervention measures, results risk of bias evaluation tools, outcome indicators, and other information. In case of disagreement, all reviewers participated in the discussions to resolve the issue.

### 2.5 Assessment of risk of bias in the included studies

The bias risk will be evaluated by 2 reviewers through Cochrane Handbook V.5.1.0 and the contents included blinding of outcome assessment, allocation sequence concealment, blinding of participants and personnel, incomplete outcome data, selective reporting, and other sources of bias. Based on these criteria, the studies will be evaluated for high, low, or unclear risk.

### 2.6 Assessment of heterogeneity

The meta-analysis will perform to use the software provided by Cochrane's official website, RevMan5.4. The heterogeneity test will be performed for all data using the I^2^ statistic. It was considered low or no heterogeneity if I^2^ < 50%; otherwise, when I^2^ > 50%, we believe that there was obvious heterogeneity. At this point, sensitivity analysis and subgroup analysis should be carried out to find the source of heterogeneity.

### 2.7 Assessment of reporting biases

When there are >10 trials in accordance with the study, the funnel charts will be used to analyze the quality of publication bias. We will conduct a test for funnel plot asymmetry using the Egger method.

### 2.8 Subgroup analysis

When there shows significant heterogeneity, we will conduct a subgroup analysis to analyze the sources of heterogeneity according to the factors affecting the results such as: sex, race, course, sample size, intervention, and outcome measurements.

### 2.9 Sensitivity analysis

In order to test the stability and reliability of the results of this study, we conducted a sensitivity analysis according to the recommendations of the Cochrane Handbook. The primary points included method quality, sample size, and missing data. Then we will perform a data analysis again by inputting missing data and changing the type of research or compare the results. If none of them had reversal changes, it indicated that the analysis results were robust and had high confidence.

### 2.10 Grading the quality of evidence

With the grading of recommendations, assessment, development, and evaluation to analyze the quality level of evidence is credible.^[14]^ We will assess the publication, heterogeneity, indirectness, imprecision, and publication bias. Finally, divide the study quality into 4 levels from high to low: high, medium, low, and very low.

### 2.11 Ethics and dissemination

The data we used were aggregated published so ethical approval was not required. This article will be published in peer-reviewed journals and presented at relevant conferences.

## 3 Discussion

CGH is a common clinical challenge in people who are 30 to 44 years old. It is thought to be pain caused by any structure innervated by the C1–C3 spinal nerves.^[15,16]^ Aseptic inflammation and nerve sensitivity caused by cervical disc lesions can aggravate the pain in a CGH. Unfortunately, it tends to recur and can significantly affect the quality of life. The primary treatment included physical therapy, exercise, and interventional procedures. Among the rest, physical therapy is considered the first line of treatment.^[17]^ Which massage therapy and acupuncture therapy are effective in treating a CGH.^[18]^ Its mechanism of action may be to release the neurotransmitter by stimulating local nerves, reduce inflammatory responses, reduce neural sensitivity, and reduce tension in the neck muscles, thus reducing pain symptoms. The combination of traditional acupuncture and massage can greatly improve treatment effectiveness, and have a significant effect on the treatment of cervical headache.^[18–20]^ However, there is no systematic review that has explored this issue. Therefore, this study will provide a high-quality synthesis to assess the effectiveness and safety of acupuncture combined with massage for treating cervical headache, and provide evidence-based medicine evidence for clinical practice.

## Author contributions

Fangfang Ding and Zhen Liu contributed to the conception of the study.

Fangfang Ding drafted and revised the manuscript.

The search strategy was developed by all the authors and will be performed by Zhen Liu and Rui Li, Chenying Wang and Rui Li extracted data from the included studies, assessed the risk of bias, and completed the data synthesis.

Yan Lu arbitrates in cases of disagreement and ensures the absence of errors.

All authors approved the publication of the protocol.

ORCID ID https://inplasy.com/inplasy-2021-12-0049/

**Conceptualization:** Zhen Liu.

**Data curation:** Rui Li, Fangfang Ding, Yan Lu.

**Formal analysis:** Zhen Liu, Chenying Wang, Rui Li.

**Funding acquisition:** Yan Lu.

**Investigation:** Fangfang Ding, Rui Li, Chenying Wang, Zhen Liu.

**Methodology:** Fangfang Ding, Zhen Liu.

**Resources:** Rui Li.

**Supervision:** Chenying Wang.

**Visualization:** Zhen Liu.

**Writing – original draft:** Fangfang Ding.

**Writing – review & editing:** Yan Lu.
